# Gut Microbiota-Derived Metabolites in Irritable Bowel Syndrome

**DOI:** 10.3389/fcimb.2021.729346

**Published:** 2021-09-23

**Authors:** Lin Xiao, Qin Liu, Mei Luo, Lishou Xiong

**Affiliations:** Department of Gastroenterology and Hepatology, The First Affiliated Hospital of Sun Yat-sen University, Guangzhou, China

**Keywords:** irritable bowel syndrome, gut microbiota, bile acids, amino acids, SCFAs, tryptophan, hypoxanthine

## Abstract

Irritable bowel syndrome (IBS) is the most common functional bowel disorder worldwide and is associated with visceral hypersensitivity, gut motility, immunomodulation, gut microbiota alterations, and dysfunction of the brain-gut axis; however, its pathophysiology remains poorly understood. Gut microbiota and its metabolites are proposed as possible etiological factors of IBS. The aim of our study was to investigate specific types of microbiota-derived metabolites, especially bile acids, short-chain fatty acids, vitamins, amino acids, serotonin and hypoxanthine, which are all implicated in the pathogenesis of IBS. Metabolites-focused research has identified multiple microbial targets relevant to IBS patients, important roles of microbiota-derived metabolites in the development of IBS symptoms have been established. Thus, we provide an overview of gut microbiota and their metabolites on the different subtypes of IBS (constipation-predominant IBS-C, diarrhea-predominant IBS-D) and present controversial views regarding the role of microbiota in IBS.

## Introduction

Irritable bowel syndrome (IBS) is a common gastrointestinal bowel disorder, characterized by recurrent abdominal pain and discomfort or alterations in bowel habits. Interestingly, women are more susceptible (1.67 times) than men (Study Group of Functional Gastrointestinal Disorders et al., 2020) to suffer from IBS. Based on different geographical regions and diagnostic criteria, the global prevalence of IBS is approximately 10–15% and is 1.4–11.5% in China. However, the proportion of IBS patients seeking clinical service in clinics is below 25% in China (Study Group of Functional Gastrointestinal Disorders et al., 2020). Based on the predominant symptoms and bowel habits, IBS patients are divided into four subtypes: constipation-predominant IBS (IBS-C), diarrhea-predominant IBS (IBS-D), mixed IBS (IBS-M), and unclassified IBS patients. The pathogenesis of IBS is associated with disordered gastrointestinal motility, abnormal intestinal secretion, visceral hypersensitivity, altered gut-brain axis, and intestinal permeability, all of which can be affected by the gut microbial community (Jeffery et al., 2020). Pittayanon et al. (2019) reported that although the role of gut microbiota in IBS pathogenesis has been gradually elucidated, it remains unclear whether microbiota dysbiosis is a cause of IBS. Investigations into gut microbial interactions between host and microbial metabolites may advance our understanding on IBS development. Owing to the heterogeneous characterization, IBS poses a significant medical burden, and considerably impact patient quality of life of patients with IBS (Enck et al., 2016; Sperber et al., 2017).

Recent studies have demonstrated that IBS symptoms are influenced by environmental factors, diet, the enteric microbial community, and host genetics and psychology (Black and Ford, 2020). However, deciphering the relevant specific signaling pathways between the gut microbiota and host remains challenging, owing to the limitations of current proposed animal models. Moreover, differences in species and host physiology have been a barrier to investigating the involvement of microbiota-derived metabolites in IBS.

Many metabolites are likely to serve as signaling molecules that influence biological functions. Alterations in metabolite production in the gut, from either host or microbiota or their interaction, may be related to the manifestation of IBS symptoms. There are mounting reports on the effects of intestinal microbial metabolites on human health. While the relationship between microbial metabolites and the development of IBS symptoms has not been clearly elucidated. Based on the extensive interaction of the co-metabolism of gut flora and the host, metabolites might provide a new approach for studying the host-microbiota system. Hence, the gut microbial metabolome may reflect the metabolic variables and clinical phenotypes, which may predict the subtypes and severity of IBS (James et al., 2020). According to the recent progress in research on the mechanism of intestinal microbial-derived IBS metabolites, further investigation into the production of gut microbiota-derived metabolites, which may in part underlie the pathophysiology of IBS, are urgently needed. Alterations and fluctuations in gut microbiota and its metabolites appear to promote development and maintenance of IBS symptoms.

## Alterations of Gut Microbiota in IBS

Human microbiota comprises trillions of microorganisms, most of which coexist in the gastrointestinal tract (Krautkramer et al., 2021). In the human gut, the vast and complex microbial community is composed of approximately 100 trillion organisms of more than 1000 different species (Tierney et al., 2019). The total number of microorganisms in the human gut is higher (100 times) than the total number of human cells. Most intestinal bacteria belong to the phyla *Bacteroidetes, Firmicutes, Actinobacteria*, and *Proteobacteria* (Lagier et al., 2015). The highest microbial biomass is found in the host cecum and proximal colon with the small intestine having a similar number of microbes as the large intestine (Krautkramer et al., 2021). Physiologically, the mucus epithelium barrier provides a foundation for commensal-microbe persistent colonization and symbiotic functions. Once the integrity of the barrier is compromised by harmful endogenous or exogenous factors, the protective effect is lost, provoking an inflammatory response and altering the gut microbial composition. A series of reports have elucidated that the loss of microbial diversity and richness is engaged in IBS pathogenesis (Jeffery et al., 2020).

Diversity (α-diversity and β-diversity) of microbes is associated with gut disorders. In a previous study, gut microbial diversity in IBS patients was significantly higher than that in the healthy control group. Several significant metabolite-microbe relationships were also revealed, including the glycine strong positive association with *Clostridium* (Zhuang et al., 2018) and homocysteine positive correlation with *Lachnospira*, *Clostridium*, and *Haemophilus* and its negative correlation with *Corynebacterium* and *Lachnospiraceae* (Zhuang et al., 2018). Previous research has shown that, among gut bacteria, the abundance of *Lachnospira* and *Clostridium* were significantly high in IBS patients (Zhuang et al., 2018), and *Clostridium difficile* has been shown to increase the risk of post-infectious IBS (Piche et al., 2007; Wadhwa et al., 2016). Moreover, a significant decrease in *Firmicutes* and an increase in *Bacteroidetes* were observed in IBS-D patients. The composition and activity of *Bifidobacterium* was low in IBS-patient stool and mucosal samples. The number of *Bacteroides* was high in IBS patients. Potential pathogen overgrowth, such as that of *Escherichia coli* and *Enterobacterium*, was also verified. However, there was no significant difference in the numbers of *Bacteroides* and *Enterococcus* between IBS patients and healthy control individuals (Zhuang et al., 2017). Many isolated archaea species were methanogens and halophiles. Moreover, patients with IBS exhibited an enrichment of bacterial taxa, such as *Enterobacteriaceae, Streptococcus, Fusobacteria, Gemella*, and *Rothia*, as well as depletion of health-promoting bacterial genera, such as *Roseburia* and *Faecalibacterium* (Sciavilla et al., 2021).

Recently, mucosal biofilms were identified as an endoscopic feature in subgroups of IBS and ulcerative colitis. The formation of mucosal biofilms is a unique growth mode of microorganisms, serving as a protective shield for bacteria. As such, biofilms can protect the bacteria against external interference and promote gene information and nutrient exchange. In fact, biofilms were detected in 57% of IBS patients, compared to controls (Baumgartner et al., 2021). Bacterial biofilms are associated with gut microbiol dysbiosis and increased levels of intestinal bile acids (Baumgartner et al., 2021). Although existing methods on fecal microbial profile or single genus were difficult to distinguish IBS patients from healthy individuals, which indicates that changes in the gut microbiota of IBS are likely to be a heterogeneous and individualized process. The presence of mucosal biofilms may contribute to the pathophysiology of IBS. Biofilms could be disrupted to alleviate functional IBS symptoms, which might offer a novel diagnosis as well as targets for treatment.

## Metabolites of Microbiota-Most Interaction in IBS

### Bile Acids

**Figure 1:**
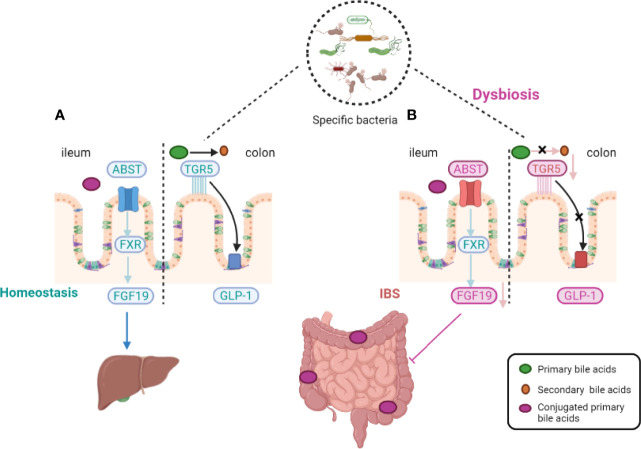
Host-microbiota interactions during bile-acid metabolism. In homeostasis, primary bile acids are converted into secondary bile acids with the assistance of intestinal flora and Takeda-G-protein-receptor-5 (TGR5) (see the figure, part **A**). Gut microbiota alterations induce an impairment in the ileal absorption of BAs, which occurs normally *via* the apical-sodium BA transporter (ASBT). This induces a decrease in the expression of nuclear Farnesoid-X receptor (FXR) and fibroblast growth factor 19 (FGF19) in intestinal epithelial cells and the abundance of colonic primary conjugated BAs. Gut microbiota dysfunction leads to a decreased transformation of primary conjugated BAs to secondary BAs in the colon, leading to defective activation of (TGR5). TGR5 of glucagon-like peptide 1 (GLP-1) activation effect was thus inhibited (see the figure, part **B**).

Bile acids (BAs), as the hub of central signals, integrate microbiota-derived signals into enterohepatic signaling and active the signaling pathways through farsenoid X receptor and G protein–coupled BA receptor 1 (GPBAR1, also called Takeda G-coupled receptor 5) (Camilleri and Vijayvargiya, 2020). BA metabolism is affected by dietary intake, environmental factors, and resident microbiota, and may be correlated with IBS (Nightingale and Lennard-Jones, 1993; Camilleri et al., 2014; Camilleri et al., 2016; Camilleri et al., 2017; Long et al., 2017). It has been verified that the levels of BAs may be associated with visceral pain and colonic transit (Dior et al., 2016). BAs in hepatocytes are generated from cholesterol by special rate-limiting enzymes. There are two pathways that regulate the expression of BAs in the liver. Approximately 75% of total BAs are produced by the classic pathway with cholesterol 7α-hydroxylase (CYP7A1), whereas the alternative BA pathway is regulated by sterol-27-hydroxylase (CYP27A1). The activity of the two rate-limiting enzymes, CYP7A1 and CYP27A1, is affected by the gut microbiota (Sayin et al., 2013). Approximately 95% of BAs are recycled *via* the hepatic circulation, controlled by fibroblast growth factor 15 and farnesoid X receptor (FXR) (**Figure 1**) (Shin et al., 2013). The second natural BA receptor is Takeda G-coupled receptor 5 (TGR5) which mediates effects of BAs on mobility and acts on enteric neurons to release serotonin. The specific relationship between the fluctuation of BAs levels and the destruction of fibroblast growth factor 15, ileal epithelial transporter, or microbial modification of the relevant metabolites remains unclear.

Primary BAs are essential for lipid/vitamin digestion and absorption (Joyce and Gahan, 2016; Zheng et al., 2017). The primary BAs chenodeoxycholic acid (CDCA) and cholic acid (CA) are synthesized in the liver from cholesterol *via* enzyme CYP7A1. The synthesis of CDCA may be facilitated by the activation of intracellular secretory channels, increase in mucosal permeability, or reduction of fluid absorption (Makishima et al., 2002). CDCA and CA are transformed into secondary BAs and deconjugated by gut microbiota and can either be passively reabsorbed and reenter the circulating BA pool or excreted in feces (Thomas et al., 2008). Unlike CDCA and CA, which are predominantly recycled, the secondary BA lithocholic acid is infrequently reabsorbed; however, it suffers further modifications by bacteria in the colon and then, is excreted (Odunsi-Shiyanbade et al., 2010). Another secondary BA, deoxycholate (DCA), is positively correlated with serotonin (5-HT) production, which is mainly regulated by Clostridia species (Yano et al., 2015). The decrease of total and primary BAs and the increase of fecal lithocholic acid are significant predictors of stool weight, frequency, and consistency. Fecal BA is a cost-effective, accurate biomarker, and it is associated with obvious bowel dysfunction in IBS-D and IBS-C patients.

In patients with chronic diarrhea or IBS-D, BA malabsorption (BAM) is positively associated with accelerated colonic transit time and influenced by gut microbiota composition (Sadik et al., 2004). In fact, a study reported that 16.9-35.3% of IBS-D patients had been diagnosed with BAM (Slattery et al., 2015). Abnormal intestinal motility results from a series of processes. First, BAs are passively absorbed by interacting with and activating TGR5 receptors on enteric neurons. Secondly, the effect of BA-induced colonic dysfunction may result from microbiota dysbiosis. In this regard, fecal BAs were related to stool characteristics and colonic transit time, and IBS-D patient’s feces with increased fecal BAs were also positively associated with *Clostridia* bacteria (Shin et al., 2013). BAM is also associated with diarrhea, caused by an increase in colonic BA concentrations due to BA insufficient recycling or overproduction (Sadik et al., 2004; Wong et al., 2012).

There are significant differences in the concentration of BAs in feces and serum between IBS-C and IBS-D patients (Wong et al., 2012). Although primary BAs are elevated in the stool of both IBS-C and IBS-D patients (Mars et al., 2020), secondary BAs are reduced in the latter group (Dior et al., 2016). A small number of BAs ultimately excreted in stool daily can be replenished through BAs *de novo* synthesis (Mars et al., 2020). C4 (7-*α*-hydroxy-4-cholesten-3-one) is positively associated with FGF19 and total BAs in IBS-D individuals, which indicates that the loss of BAs in feces leads to an increase in BA production. Moreover, patients with IBS-D have a higher proportion of *Escherichia coli* and decreased *Clostridium leptum* and *Bifidobacterium* (**Table 1**) (Duboc et al., 2012; Dior et al., 2016; Wei et al., 2020). However, a recent study showed that 24.5% IBS-D patients presented with a higher level of total BAs and *Clostridia* bacteria (Zhao et al., 2020). Additionally, fecal BAs and serum C4 levels are positively associated with Clostridia bacteria, which is negatively correlated with serum FGF19 (Zhao et al., 2020). Clostridia-rich microbiota may enhance BA synthesis and excretion in IBS-D patients by reducing gastrointestinal transit time and increasing fecal water content. These finding suggest that *Clostridia* bacteria have potential as a biomarker for bile acid diarrhea or IBS-D and as a target for therapy.

**Table 1:** Gut microbiota-derived metabolites and associated genera or species in IBS.

Metabolites	Pathogenesis	Species	Reference
Bile acids	GI mobility and gut permeability	*Bifidobacterium* and *Clostridium* *Escherichia coli*	Duboc et al., 2012; Dior et al., 2016; Wei et al., 2020
SCFAs	visceral hypersensitivity and inflammation	*Veillonella, Lactobacillus, Lachnospira, Clostridium* spp.*, Bifidobacterium* spp.*, Veillonella* spp.*, Clostridia, Bifdobacteria, Ruminococccaceae* and *Erysipelotrichaceae*	Louis et al., 2010; Canani et al., 2011; Ringel-Kulka et al., 2015; Barbara et al., 2016; Rajilić-Stojanović et al., 2015; Farup et al., 2016; Morrison and Preston, 2016
Acetate		*Bacteroides* spp. *and Veillonella* spp.	Barbara et al., 2016
Propionate		*Fecalibacterium prausnitzii, Roseburia* spp.*, Eubacterium rectale, Eubacterium hallii, and Coprococcus comes*	Macfarlane and Macfarlane, 2003; Louis et al., 2010; Vital et al., 2017
Butyrate			
Tryptophan	gut permeability	*Lactobacillus*	Vujkovic-Cvijin et al., 2013; Agus et al., 2018; Sun et al., 2019
Neurotransmitters			
GABA	Visceral pain, inflammation, and visceral hypersensitivity	*Lactobacillus brevis* and *Bifidobacterium dentium*	Mazzoli and Pessione, 2016
Dopamine	Inflammation, visceral pain, GI mobility, and psychological factors	*Escherichia coli*, *Klebsiella pneumoniae*, *Pseudomonas aeruginosa*, *Shigella sonnei* and *Staphylococcus aureus*	Strandwitz, 2018
5-HT	Changes in ENS and gut–brain axis, visceral pain, and visceral hypersensitivity	*Corynebacterium* spp., *Streptococcus* spp. and *Enterococcus* spp.	Walther et al., 2003
Vitamins			
Vitamin D	Inflammation and gut permeability	*Salmonella typhimurium*	Wu et al., 2010
Vitamin B6	Inflammation	*Actinobacteria*, *Bacteroidetes*, and *Proteobacteria* phyla	Lagier et al., 2015
Hypoxanthine		*Lachnospiracea* strains, *Barnesiella* and *Prevotella*	Zhao et al., 2013; Mars et al., 2020

SCFAs, short-chain fatty acids; GABA, γ-aminobutyric acid; 5-HT, serotonin; GI mobility, gastrointestinal mobility; ENS, enteric nervous system.

BA content in stool samples is significantly high in animal-based diets compared with plant-based diets, owing to the difference in cholesterol (David et al., 2014). The concentration of BAs is highly correlated with dietary patterns, genes of microbial enzymes, and metabolites. Thus, BAs fluctuations could offer a possible approach to understand the mechanisms of the production and development of IBS.

## Short-Chain Fatty Acids

Short-chain fatty acids (SCFAs) are critical fermentation products of the gut microbiota and related to the pathogenesis of various gastrointestinal disorders such as IBS. Approximately 95% of SCFAs are acetate, propionate, and butyrate (at a molar ratio of 3:1:1) that are produced from carbohydrates in the colon and are correlated with the gut microbiota. SCFAs can affect the integrity of the intestinal mucosa, glucose and lipid metabolism, immune system, and inflammatory responses. The specific effects of SCFAs are mediated by several mechanisms, including selective activation of the G-protein-coupled receptors family (GPCR), free fatty-acid receptors (FFARs), or epigenetic effects. Altered fecal SCFAs are associated with relative abundance of bacteria, including *Clostridia*, *Bifdobacteria, Ruminococccaceae* and *Erysipelotrichaceae* (Rajilić-Stojanović et al., 2015).

Butyrate is synthesized by *Faecalibacterium prausnitzii*, *Roseburia* spp., *Eubacterium rectale*, and *Eubacterium hallii* through alternate pathways using exogenous acetate (Louis et al., 2010; Vital et al., 2017). Butyrate is also produced *via* classical pathways utilizing acetate, sugars, and amino acids by other bacteria, such as *Coprococcus comes* (Macfarlane and Macfarlane, 2003). There are three pathways to produce propionate, including the acrylate, succinate, and the propanediol pathways. The most common is the succinate pathway, adopted by *Bacteroides* spp. and *Veillonella* spp. (Barbara et al., 2016). The production pathways of acetate are the pyruvate decarboxylation to acetyl-CoA and Wood-Ljungdahl pathways. It was reported that several strains of *Lachnospira* produce lactate and acetate, causing constipation symptoms linked to mucin secretion (Canani et al., 2011). Some of the most predominant microbiota producing acetate include *Clostridium* spp. and *Bifidobacterium* spp. (Louis et al., 2010; Barbara et al., 2016; Morrison and Preston, 2016); *Clostridiales sp.* exhibit different distributions in patients with IBS-C and IBS-D (Gargari et al., 2018). SCFAs are utilized to provide energy to different organs, such as butyrate in the colon and propionate in the liver, by gluconeogenesis. Acetate and propionate are associated with fatty acid and energy regulation in the liver (Morrison and Preston, 2016). Therefore, the relative and proper proportions of specific SCFAs may be important for maintaining homeostasis. The alterations of SCFA composition and concentration are apparent in IBS patients (Tana et al., 2010; Farup et al., 2016). Reduction of the butyrate concentration in IBS patients may cause energy salvage in the colon and IBS symptoms (Farup et al., 2016). However, no difference was found in fecal propionate, butyrate, and lactate levels between control and IBS patients, and the levels of propionic and butyric acid in serum of IBS-D patients was high. A different study showed that the concentration of SCFAs and the relative abundance of *Veillonella* and *Lactobacillus* in fecal samples of IBS participants was high (Ringel-Kulka et al., 2015; Farup et al., 2016). *Lactobacillus* can prominently produce lactic and acetic acids, whereas *Veillonella* can convert lactic acid into acetic acid and propionic acid (Tana et al., 2010). In this context, there is a positive correlation between fecal SCFA concentration and IBS-symptom severity, indicating that there may be an association between metabolite production and gut microbiota (Tana et al., 2010).

Although the association between SCFAs and IBS patients is controversial in the literature, as a series of studies report both high and low SCFA concentrations (Treem et al., 1996; Tana et al., 2010), the specific SCFA profiles differed between IBS subtypes resulting in an accelerated transit rate in IBS-D patients with hypermotility with stimulatory and inhibitory effects.

## Amino Acids

Proteins are mainly metabolized in the small intestine, and approximately 5 to 10% of dietary proteins (as proteins and peptides) is absorbed (Mahé et al., 1992; Evenepoel et al., 1999). Proteins are catabolized into amino acids, which can enter the portal circulation as vital intermediates of the host–microbe complex interaction. The gut microbial assimilation process of amino acids is largely regulated by carbohydrate substrates and luminal contents (Smith and Macfarlane, 1996; Ratzke and Gore, 2018). In addition, it is clear that the gut microbiota, involved in the energy metabolism of host proteins and peptides, will produce various bioactive compounds, including tryptophan, SCFAs, branched-chain fatty acids, ammonia, phenols, indoles, and amines (Ratzke and Gore, 2018). Therefore, the remainder of this paper focuses on a list of vital bioactive amino acid derivatives with known effects and potential mechanisms in IBS patients.

## Tryptophan

Tryptophan is a vital aromatic amino acid that can be obtained from dietary intake. Tryptophan is a free and the most chemically complex amino acid, making it an optimal molecule for extensive transformations. As for its role in protein synthesis, tryptophan is a precursor of crucial metabolites, such as 5-HT, which plays an essential role in functional gastrointestinal diseases (Berstad et al., 2014). The two main pathways in the host to metabolize tryptophan obtained from dietary intake are the kynurenine and 5-HT routes, which metabolize tryptophan to the vitamin niacinamide, 5-HT, or melatonin (Berstad et al., 2014; Heitkemper et al., 2016). Thus, tryptophan may be important to regulate the balance between the vitamin niacinamide, 5-HT, and melatonin because the functions of these metabolites are different (Clarke et al., 2009; Clarke et al., 2012; Berstad et al., 2014; Heitkemper et al., 2016).

The gut microbes in the third pathway can metabolize tryptophan into several molecules, such as indole and other derivatives compounds, with some of them acting as ligands to bind the aryl hydrocarbon receptor (AhR) (Williams et al., 2014; Agus et al., 2018). A recent study reported that the symptoms of a metabolic disorder were associated with a reduced microbial capacity to transform tryptophan into AhR agonists (Liu et al., 2021). The AhR pathway is accompanied by a decrease in glucagon-like peptide-1 (GLP-1) and Interleukin-22 (IL-22), resulting in changes in the intestinal permeability and lipopolysaccharide in functional gastrointestinal diseases. In addition, AhR agonists or *Lactobacillus* regulation can be an available treatment to reverse metabolic disorders (Vujkovic-Cvijin et al., 2013; Agus et al., 2018; Sun et al., 2019).

The concentration of histidine, lysine, glutamine, proline, and glutamic acid are different between patients with IBS and inflammatory bowel disease. Ornithine, as the only amino acid in low levels in IBS patients, is essential for the urea cycle (Keshteli et al., 2019). Glutamine is associated with energy supply of intestinal epithelial cells; owing to its low levels, it may play a key role in IBS symptoms. Existing data also suggest the remarkable potential of tryptophan-derived aryl hydrocarbon receptor agonists, indole derivatives on lumen equilibrium (Zhang et al., 2021). Specifically, microbiota derived-tryptophan as therapeutic interventions have potential to promote proinflammatory or anti-inflammatory responses in the gut (Zhou et al., 2019). Therefore, further research is needed to investigate the potential role and clinical relevance of amino acid metabolism in IBS.

## Neurotransmitters

Serotonin (5-HT), as a key transmitter in neuronal signaling, provides insights into the complex interactions of the gut-brain-microbiota axis (Cremon et al., 2011). Meanwhile, an increased abundance of 5-HT has been reported in the blood of IBS patients (Luo et al., 2021). 5-HT is produced from tryptophan *via* the two-step pathway. Tryptophan hydroxylase (TPH) is a rate-limiting enzyme with two isoforms (Tph1 and Tph2). Studies have also shown that the gut microbiota could induce the transcription of Tph1 and Tph2 and subsequent 5-HT production (Yano et al., 2015). By using a metabolites-based framework, approximately 20% of the microbial genome has the potential for 5-HT synthesis (Valles-Colomer et al., 2019). Tph1 is mainly expressed in enterochromaffin cells of the gut epithelium, whereas Tph2 is mainly produced in serotonergic neurons of the central and enteric nervous systems (Walther et al., 2003). The intestinal resident microorganisms, including *Corynebacterium* spp., *Streptococcus* spp., and *Enterococcus* spp., can also trigger IBS symptoms (Walther et al., 2003). Enteric 5-HT is vital to regulate gut peristalsis and secretion, inflammation and development of neurons and interstitial cells of Cajal. It is assumed to be related to pain, sensitivity, and reflexes *via* the activation of enterochromaffin and enteroendocrine cells (Walther et al., 2003; Mawe and Hoffman, 2013). A study has shown that decreased uptake of 5-HT was linked to the deletion of a base fragment, especially in IBS-D patients (Pata et al., 2002; Atkinson et al., 2006). Enterochromaffin cells, mast cells in mucosal layers, and 5-HT level were significantly up-regulated in IBS patients compared with healthy individuals (Cremon et al., 2011). The degree of visceral pain and hypersensitivity in patients with IBS is related to the release of 5-HT (Cremon et al., 2011). The up-regulated 5-HT level leads to increased hypersensitivity in IBS patients associated with nerve-sensing mechanisms. 5-HT reuptake transporter (SERT), as the recycling mechanism for 5-HT, may affect the metabolism in IBS individuals because of its polymorphisms, although there are controversies about the potential relationship between 5-HT, the *SERT* gene, and IBS (Gershon and Tack, 2007; Cremon et al., 2011; Makker et al., 2015).

Dopamine (DA), a precursor for adrenaline and noradrenaline, is decreased in individuals with IBS (Dunlop and Nemeroff, 2007). It has been proven that DA can control chronic pain (Mitsi and Zachariou, 2016) and psychological disorders, and it can regulate intestinal inflammation by inhibiting the NLRP3 inflammasome (Yan et al., 2015; Li et al., 2020). The dopamine D2 receptor antagonist has been applied to treat IBS patients to improve gastrointestinal motility (Tack et al., 2006). The dopamine D5 receptor may play an important role in increasing the permeability of duodenal epithelial cells and protecting the colonic mucosa (Li et al., 2019). Moreover, several bacterial strains are highly correlated with alterations of dopaminergic pathways for growth and metabolism, including *E. coli, Klebsiella pneumoniae, Pseudomonas aeruginosa, Shigella sonnei*, and *Staphylococcus aureus* (Strandwitz, 2018). In conclusion, the dysfunction of the dopaminergic signaling pathway may underline the pathogenesis of IBS.

Compared with healthy controls, the level of γ-aminobutyric acid (GABA), as an inhibitory transmitter that regulates peripheral afferent nerve visceral pain, is reduced in IBS-D individuals (Ligaarden and Farup, 2011). Glutamate decarboxylase, a rate-limiting enzyme, can metabolize glutamate to GABA. Mazzoli and Pessione (2016) reported several strains that regulate the hypersensitivity of the intestinal viscera of IBS rats, such as *Lactobacillus brevis* and *Bifidobacterium dentate*, can also produce GABA (γ-aminobutyric acid). Reduced GABA levels can lead to depression and anxiety disorders in the pathology of IBS-D through low-grade inflammation (Potts et al., 2016). It has been reported that the lack of glutamic acid decarboxylase (GAD), which controls GABA synthesis, can cause neurological diseases. Enhancing GABA inhibition could alleviate depression, anxiety, and chronic pain in patients with IBS (Adeghate and Ponery, 2002; Aggarwal et al., 2018). Accordingly, gut microbiome-mediated GABA may be correlated with the pathogenesis of IBS.

## Vitamins

The intestinal microbiota actively affects the one-carbon metabolism and the utilization of vitamins, especially vitamin D and B6 (pyridoxine), in IBS patients (Ligaarden and Farup, 2011). Vitamins are directly obtained from dietary intake or biosynthesized in the host. However, vitamins produced only by the host may not meet the effective function of cellular processes. As compared to healthy individuals, *Firmicutes* are often increased in IBS patients; *Firmicutes* is the only phylum analyzed that presents eight key-vitamin synthesis pathways (Krogius-Kurikka et al., 2009; Jeffery et al., 2012; Magnúsdóttir et al., 2015).

Vitamin D plays a vital role in maintaining enteric homeostasis. However, the level of vitamin D in the serum of IBS patients is reduced (Miura et al., 2021). Lack of vitamin D and/or its receptor (VDR) can lead to a series of proinflammatory cytokines, such as TNF-α and IFN-γ, which can weaken the mucosal barrier function, increasing permeability (Bruewer et al., 2006; Reich et al., 2014). The expression of VDR in the duodenum of patients with IBS is increased (Wu et al., 2010). VDR is associated with the intestinal barrier function, immune responses, and colitis, and it is also vital for the activation of NFκB and the expression of tight junction proteins in the intestinal flora (Ilmer et al., 2014; Chen et al., 2015). Additionally, 1α,25-dihydroxyvitamin D_3_[1,25(OH)_2_D_3_], an active form of vitamin D, can directly interact with the intestinal flora and resist the lipopolysaccharides or TNF-α (Ilmer et al., 2014; Chen et al., 2015). The level of vitamin D is positively correlated with the level of 1,25(OH)_2_D_3_ in the serum, but VDR expression can be down-regulated by pathogenic species such as *Salmonella typhimurium* in the intestine (Wu et al., 2010). Vitamin D affects enteric cells through aberrant signaling pathways and interactions with its cellar receptor (VDR), contributing to its antimicrobial functions with several antimicrobial peptides such as lysozyme and β-defensin-2 (Hewison, 2011). Thus, vitamin D and/or VDR deficiency might play an important role in the pathogenicity of IBS owing to their role in intestinal barrier functions and low-grade mucosal inflammations. However, in a recent study, no benefit was reported following vitamin D supplementation on IBS symptoms nor on quality of life (Williams et al., 2021). Thus, no evidence exists to support the application of vitamin D supplementation for IBS treatment as no clear relationship between vitamin D levels and symptomology has been demonstrated.

Vitamin B6, a water-soluble vitamin (Rosenberg et al., 2017), may play a vital role in IBS and participate in inflammatory conditions (Ligaarden and Farup, 2011). Pyridoxal 5-phosphate is a bioactive form of vitamin B6 that is characterized by a series of coenzymes that may be involved in cellular metabolism, including amino acid, BA and lipid functions (Cellini et al., 2020). Vitamin B6 might be involved in the inflammation-mediated pathogenesis of IBS. The levels of vitamin B6 are linked to a high IBS-symptom score, and the symptoms can be alleviated by increased vitamin B6 intake. The synthesis of vitamin B6 is influenced by a variety of bacterial species (Huang et al., 2010; Rosenberg et al., 2017), including *Actinobacteria, Bacteroidetes*, and *Proteobacteria.* Interestingly, *Firmicutes* was found to have a low ability to synthesize vitamin B6 (LeBlanc et al., 2013).

Differences in vitamin-synthesizing ability of different microbial species might identify the potential mechanism for vitamin variation between healthy individuals and IBS patients. Hence, it is interesting to investigate microbiota-mediated changes in vitamin D and B6 for effective diagnosis and management of IBS patients.

## Hypoxanthine

The purine nucleobase hypoxanthine, as an available substrate for efficient purine nucleotide biogenesis, is a significant host-microbial co-metabolite (Mars et al., 2020). It has been reported that the fecal hypoxanthine abundance in mice is strongly associated with the genus *Barnesiella* and *Prevotella* (Zhao et al., 2013). Moreover, hypoxanthine is associated with energy metabolites and DNA replication and serves as a source of energy for intestinal epithelial cells and enhance the ability of intestinal cellular barrier recovery by decreasing oxygen consumption (Lee et al., 2018; Lee et al., 2020). Additionally, the metabolism of hypoxanthine plays an important role in regulating the production of H_2_O_2_ and superoxide anions in IBS (Mete et al., 2013). The *de novo* synthesis of purines in intestinal epithelial cells is limited, requiring five ATP molecules for production of one purine molecule. Thus, purine metabolism primarily depends on the salvage pathway, as a resource-conserving alternative (Biaggioni et al., 2015). The purine salvage pathway represents the complement of nucleotide metabolism and is vital for the biosynthesis of hypoxanthine. Dehydrogenase/oxidase (XO) and hypoxanthine phosphoribosyl transferase (XPRT) are important enzymes in hypoxanthine metabolism. Hypoxanthine phosphoribosyl transferase (HPRT) transforms xanthosine into xanthine through the purine salvage pathway. XO is a xanthine and hypoxanthine catalytic enzyme with poor specificity through the purine *de novo* pathway.

**Figure 2:**
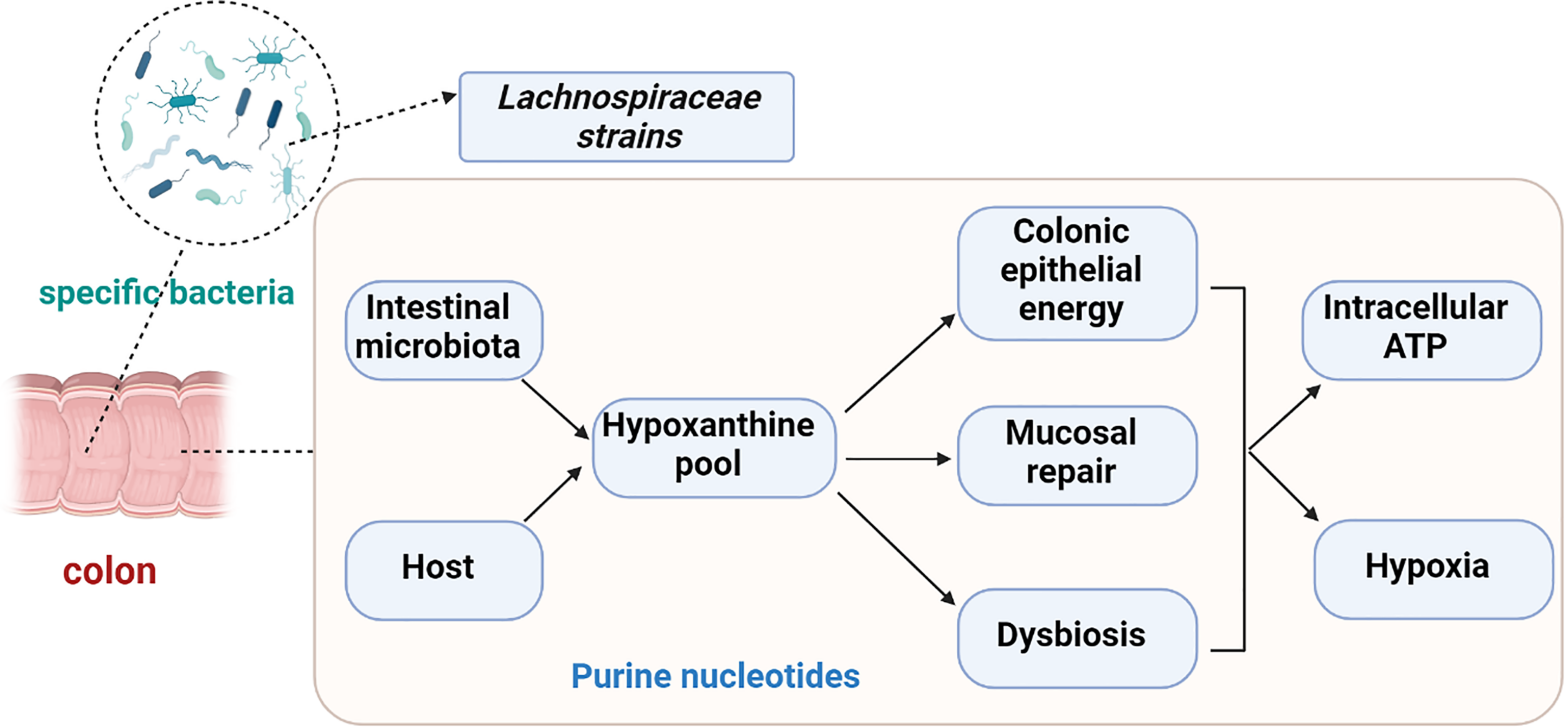
Purine starvation in colonic epithelium as a potential novel mechanism underlying IBS due to depletion hypoxanthine pool. Hypoxanthine might cause irritable bowel syndrome (IBS) symptoms *via* colonic epithelial energy, the capacity for mucosal repair or dysbiosis. Decreased fecal hypoxanthine abundance were most strongly associated with *Lachnospiraceae* strains.

Recently, reduced amounts of hypoxanthine have been reported in stool samples of patients with IBS-C and IBS-D (Mars et al., 2020). Moreover, the levels of XO and XPRT were elevated in IBS-C relative to healthy controls. Purine nucleoside phosphorylase (PNP) is significantly increased in IBS-C and IBS-D patients, however, it is negatively correlated with hypoxanthine concentration (Mars et al., 2020). In both IBS subtypes, the expression of the human XO gene is up regulated. Additionally, gut microbiota reportedly utilize hypoxanthine as a substrate, making it a cross-feeding substrate. Hence, the reduction of fecal hypoxanthine could reflect the production of hypoxanthine by the gut microbiota in IBS patients. That is, the decreased fecal hypoxanthine abundance in IBS patients may result from the increased capacity for its utilization and breakdown, particularly by *Lachnospiraceae* strains. The ensuing purine starvation was identified as a potential novel mechanism underlying IBS (**Figure 2**) (Mars et al., 2020).

Hypoxanthine is a host-microbial co-metabolite that can be affected by both microbial and host metabolism. Thus, microbiota-derived purines (especially hypoxanthine) appear to represent vital contributors to, and critical substrates for, colonic barrier function, capacity for mucosal repair, and a healthy microbiota, warranting further exploration and investigation in IBS patients.

## Conclusions

The underlying mechanisms regulating the interaction between the gut microbiota and host still remain unclear in IBS. It is difficult to achieve consensus on the relationships between specific microbiota-derived metabolites and subtypes of IBS. New evidence suggests that research and clinical practices should move away from relying on symptoms and experiences as a diagnostic and results-based tool. Understanding variations and fluctuations in the concentrations of host or microbiota-derived metabolites that can be used to infer processes contributing to the symptoms and severity of IBS will provide important new insights for functional gastrointestinal disorders (FGDs) research.

In this review article, we indicate the important microbial metabolites in the context of host physiology in patients with different IBS subtypes. We found that the abundance of microorganisms and their corresponding metabolites in IBS-C and IBS-D differ, thereby providing a new avenue for the diagnosis and treatment of different IBS subtypes in the future. These microbiota derived-metabolites, such as BAs, SCFAs, vitamins, amino acids, 5-HT, and hypoxanthine, can be produced directly by bacteria, or from dietary or relevant substrates. Fluctuations and alterations in the levels of metabolites produced by the host or microbiota provide insights into their interations during IBS. Moreover, SCFAs, dose-dependently, which effects dependent on the specific SCFA, segment of the colon animal species and experimental models, may impact IBS-D with increased transit rate. Moreover, low levels of hypoxanthine may be associated with colonic epithelial energy and capacity for mucosal repair with hypoxia. Purine starvation has been identified as a potential novel mechanism underlying IBS with lower fecal hypoxanthine abundance in IBS-C and IBS-D. Additionally, mucosal biofilms are an endoscopic feature of IBS and are associated with bacterial and BA metabolites dysbiosis.

Microbiota-derived metabolites undoubtedly plays a role in IBS severity; however, future research must include supplement clinical studies that aim to determine the underlying mechanisms. For example, intraluminal butyrate supplementary could increase colonic transit and apply into the therapy of IBS in animal models, but its effects on IBS patients are still not clear. And how we can alleviate the prevalence of IBS worldwide. For advancements to be made, investigations that undertake special metabolites interventions should be followed by a thorough analysis of the gut microbiota. Critically, this will accommodate not just a better understanding of the epidemiology of IBS but also recommendations for managements of microbiota-derived metabolites to alleviate IBS symptoms. Recommendations on metabolites based on studies lacking mechanism evidence may result in the adoption of metabolites therapy, leading to beneficial results, but equally may be detrimental after long-term adherence owing to lack of understanding of other biological mechanisms.

## Author Contributions

LX and QL conceived the study and drafted the manuscript. LSX designed and edited the manuscript. ML contributed to the collection of relevant studies. All authors contributed to the article and approved the submitted version.

## Funding

This study was supported by the National Natural Science Foundation of China awarded to LS Xiong (Grant number 81970471).

## Conflict of Interest

The authors declare that the research was conducted in the absence of any commercial or financial relationships that could be construed as a potential conflict of interest.

## Publisher’s Note

All claims expressed in this article are solely those of the authors and do not necessarily represent those of their affiliated organizations, or those of the publisher, the editors and the reviewers. Any product that may be evaluated in this article, or claim that may be made by its manufacturer, is not guaranteed or endorsed by the publisher.
